# Contrast-free functional MRI for assessing renal involvement in patients with ANCA-associated vasculitis

**DOI:** 10.1186/s12882-026-04981-3

**Published:** 2026-04-30

**Authors:** Marie Scheuer, Anna Kernder, Isabell Haase, Sara Bokonjic, Thomas Andreas Thiel, Eric Bechler, Charlotte Böttger, Helena Anne Peters, Gerald Antoch, Jörg H. W. Distler, Matthias Schneider, Alexandra Ljimani

**Affiliations:** 1 Department of Diagnostic and Interventional Radiology Dusseldorf, Medical Faculty, University Dusseldorf, D-40225 Dusseldorf, Germany; 2 Department of Rheumatology, Medical Faculty, University Hospital Düsseldorf, Heinrich Heine University, Dusseldorf, Germany; 3 Hiller Research Center, Medical Faculty of Heinrich, University Hospital Düsseldorf, Heine University, Dusseldorf, Germany; 4 Rheumazentrum Ruhrgebiet Herne, Ruhr-University Bochum, Herne, Germany; 5 III. Department of Medicine, University Medical Center Hamburg-Eppendorf, Martinistraße 52, 20246 Hamburg, Germany; 6 Department of Rheumatology and Immunology, Klinikum Bad Bramstedt, Bad Bramstedt, Germany; 7 Core Facility for Magnetic Resonance Imaging, Medical Faculty and University Hospital Düsseldorf, Heinrich-Heine-University Düsseldorf, Düsseldorf, Germany; 8 CARID Cardiovascular Research Institute Düsseldorf, University Hospital Düsseldorf, Heinrich-Heine-University, Dusseldorf, Germany

**Keywords:** Vasculitis, AAV, mpMRI, Kidney

## Abstract

**Background:**

Early detection of renal involvement in ANCA-associated vasculitis (AAV) is crucial, as functional changes often precede anatomical damage. Current diagnostic standards, such as the measurement of serum creatinine, renal biopsy and urinary analyses have limitations due to delayed detection and lack of specifity. Functional renal MRI (fMRI) techniques, including diffusion-weighted imaging (DWI), diffusion tensor imaging (DTI), arterial spin labeling (ASL) and blood oxygenation level dependent (BOLD) offer promising non-invasive alternatives for assessing renal function in AAV. The aim of this study was to evaluate the feasibility of non-invasive assessment of renal changes associated with AAV using mpMRI (multiparametric MRI).

**Methods:**

This study evaluated 7 patients and 10 healthy controls: patients with rapidly progressive glomerulonephritis (RPGN) due to AAV (*n* = 3), AAV patients without clinical signs of renal involvement (*n* = 4), and healthy controls (*n* = 10). All participants underwent functional renal MRI. Key parameters, including the apparent diffusion coefficient (ADC), fractional anisotropy (FA), and ASL-based renal perfusion and T2* parameter maps, were acquired and analyzed.

**Results:**

The following differences in renal imaging parameters were observed between RPGN patients and healthy controls: RPGN patients showed reduced ADC values in the renal medulla and increased FA values compared to controls. Additionally, ASL values in the renal cortex were lower in RPGN patients. T2* values were lower in RPGN patients compared to the healthy control group in the cortex, and higher in the medulla. Patients with AAV without confirmed renal involvement also showed alterations in ADC, T2* and FA values compared to healthy controls.

**Conclusion:**

Our findings indicate that mpMRI parameter might detect renal changes in AAV. Therefore, mpMRI might offer novel opportunities for non-invasive detection of disease-associated changes.

## Introduction/background

 ANCA-associated vasculitis (AAV) is a group of rare, life-threatening autoimmune disorders characterized by necrotizing inflammation of small to medium blood vessels and the presence of anti-neutrophil cytoplasmic antibodies (ANCA) [1]. The three principal subtypes are granulomatosis with polyangiitis (GPA), microscopic polyangiitis (MPA), and eosinophilic granulomatosis with polyangiitis (EGPA), each distinguished by clinical features such as granulomatous inflammation in GPA and eosinophil infiltration in EGPA [2].

ANCAs against proteinase 3 (PR3-ANCA) are most commonly found in GPA, while myeloperoxidase ANCAs (MPO-ANCA) predominate in MPA and EGPA.

Vasculitis encompasses a range of complex illnesses that can affect any organ system, posing significant diagnostic and management challenges due to overlapping and varying symptoms. Vasculitis can also manifest as single-organ vasculitis (SOV), characterized by inflammation of blood vessels within a single organ, such as the kidney [1].

In rare cases, with an incidence of 7 per 1,000,000, AAV can result in rapidly progressive glomerulonephritis (RPGN) [3].

Typically, renal involvement becomes symptomatic through acute kidney injury with proteinuria and erythrocyturia. The gold standard to confirm a renal involvement remains the invasive renal biopsy [4].

Given the risks associated with biopsy [5], an alternative method for diagnosing renal involvement in vasculitis is essential. Non-invasive functional MRI presents a promising option. Functional renal MRI have shown its potential to reflect renal involvement in different pathological conditions as diabetic kidney disease (DKD) and chronic kidney disease (CKD).

The apparent diffusion coefficient (ADC) measures restricted water molecule motion in renal tissue, influenced by structural features like cell membranes and the interstitial matrix [6]. Reduced estimated glomerular filtration rate (eGFR) and fibrosis in chronic renal dysfunction hinder water diffusion, leading to lower ADC values. Several studies have shown a positive correlation between ADC and GFR [7].

To characterize tissue microarchitecture by quantifying anisotropy and spatial diffusion dependence, diffusion encoding in multiple directions is required. This is achieved with diffusion tensor imaging (DTI), which necessitates at least six distinct signal encoding directions. DTI enables the calculation of FA (fractional anisotropy), reflecting tissue anisotropy [7]. Studies have already demonstrated that FA alterations are possible in kidney diseases. Liu et al. reported that DTI is valuable for the noninvasive assessment of renal function and pathology in patients with CKD. A decrease in FA was associated with glomerular lesions, tubulointerstitial injuries, and eGFR decline [8].

Approximately 25% of cardiac output circulates through the kidneys, making renal perfusion essential for nutrient and oxygen supply as well as glomerular filtration. Renal ischemia plays a critical role in acute kidney injury (AKI) and accelerates CKD progression [9]. Early assessment of renal perfusion is crucial for predicting, slowing, or preventing further deterioration. Arterial spin labeling (ASL) is a non-invasive MRI technique that uses magnetically labeled arterial blood protons as an endogenous tracer to assess tissue perfusion without exogenous contrast agents [10]. Many studies report reproducibility of renal perfusion by ASL and lower ASL values in CKD patients compared with healthy subjects, which correlates with eGFR [10–16].

Blood oxygenation level dependent (BOLD) MRI utilizes changes in blood oxygenation to create contrast, as oxygen saturation alters the magnetic properties of hemoglobin (Hb) [17]. Fully oxygenated Hb is diamagnetic, while deoxygenated Hb is paramagnetic, affecting the transverse decay time (T2*) or the rate constant (R2*). Studies have also shown that R2* values are positively correlated with kidney function and inversely correlated with the eGFR [18]. Also recent studies have reported that the evaluation of oxygenation in renal cortex by BOLD-MRI can predict the progression of renal function [19].

The aim of this pilot study was to evaluate the feasibility of non-invasive assessment of renal changes associated with AAV using multiparametric MRI (mpMRI).

## Methods

### Study population/study design

This was a prospective, cross-sectional pilot study. Patient recruitment occurred between November 2020 and October 2023 after ethical approval. All AAV patients were enrolled consecutively. Inclusion criteria were a confirmed diagnosis of AAV and availability of renal MRI. Exclusion criteria included prior renal transplantation and contraindications to MRI.

The study was approved by the local ethics committee, and written informed consent was obtained from all study participants.

Seven AAV patients were enrolled to assess the potential clinical relevance of renal mpMRI, including patients with biopsy-proven rapidly progressive glomerulonephritis associated with AAV (*n* = 3) and patients with diagnosed vasculitis without confirmed renal involvement (*n* = 4). In addition, ten healthy volunteers without any history of kidney disease, diabetes, vascular disease, prior renal surgery, or systemic diseases potentially affecting the kidneys were included.

### MR imaging

Multiparametric, functional MRI measurements were performed on a clinical 3T MRI scanner (Magnetom Prisma, Siemens AG, Healthineers, Erlangen, Germany) without the use of contrasting agents.

Functional renal sequences were performed according to consensus-based recommendations for functional renal imaging by PARENCHIMA project [20].

For each patient, MRI acquisition began with a T2-weighted HASTE (Half-Fourier Acquisition Single-Shot Turbo Spin-Echo) sequence, providing an anatomical overview of the kidneys.

This was followed by diffusion-weighted imaging (DWI) and DTI using a coronal echo-planar imaging (EPI) sequence. These techniques allowed for the assessment of water diffusion in renal tissue, enabling the calculation of the ADC and FA. The EPI sequence was acquired with 15 slices of 5 mm thickness, a field of view (FOV) of 400 × 400 mm, and Repetition Time/ Echo Time (TR/TE) of 3000/78 ms, using b-values of 0, 50, 400, 800 s/mm² with 6 diffusion encoding directions. DWI data acquisition adhered to the recommendations outlined by Ljimani et al. [21].

Renal perfusion was assessed using arterial spin labeling (ASL) with a paracoronal FAIR-TrueFISP (flow-sensitive alternating inversion recovery with true fast imaging with steady precession) sequence. This sequence acquired an M0-image followed by averages of alternating “label and control” images, 1 section; TR/TE of 5/2.5 ms, an inversion time (TI) of 1.2 s, a slice thickness of 8 mm, and an FOV of 400 × 400 mm [10].

Renal oxygenation was evaluated using BOLD MRI with a coronal T2*-weighted multi-echo gradient-echo (GRE) sequence. The sequence included 6 echoes, TR 100 ms and TE 2.46, 4.92, 7.38, 9.84, 12.30, 14.75 ms. Images were acquired with a slice thickness of 5 mm, three slices with a 1 mm gap, a matrix of 256 × 256, and an FOV of 396 × 399 mm [20].

### Image and statistical analysis

For ASL post-processing an in-house developed software “stoketool” was used. All other parameter maps were generated using in-line post-processing.

**Fig. 1:**
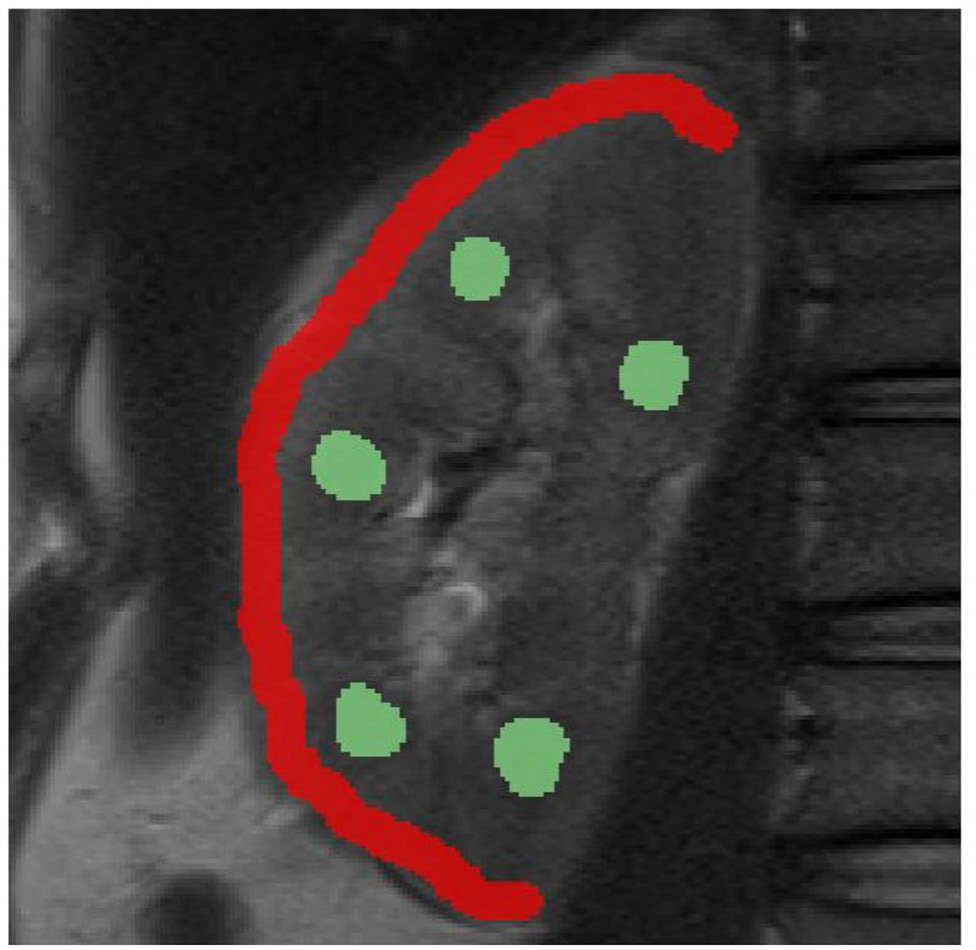
Representative example of region-of-interest (ROI) placement in the renal cortex and medulla. For statistical analysis, one continuous ROI was positioned in the renal cortex (red), whereas three to five small ROIs were placed in the renal medulla (green) for each subject

ROI (Region of Interest) based analysis were performed using the clinic’s PACS (Picture Archiving and Communication System) for each acquired sequence. ROIs were placed independently by two radiologists (M.S., 5 years of experience; A.L., 10 years of experience), both trained in renal MRI analysis. ROI positioning was performed in accordance with published consensus-based technical recommendations, using a thin, continuous ROI covering the entire renal cortex and multiple small ROIs within the medulla for each kidney [12] (Fig. 1).

Statistical analysis was performed using Microsoft Excel (Version 16.104, Microsoft Corporation, Redmond, WA, USA). Due to the low number of patients included in this study, statistical analysis was only performed for healthy control compared to all patients and not individual patient groups. Analyses were performed using Wilcoxon-Mann-Whitney u-test, and a p-value < 0,05 was considered statistically significant. Furthermore, results of different patient groups were presented descriptively to illustrate observable differences between the groups.

## Results

### AAV patients: demographics and clinical parameters

The mean age of the study cohort was 63.8 ± 11 years. Patients with RPGN due to AAV showed impaired renal function, with eGFR ranging from 12 to 58 ml/min and serum creatinine levels between 0.9 and 4.16 mg/dl (Table 1). Most of these patients also showed relevant proteinuria (up to 839 mg/l) and marked hematuria (up to 1204 cells/µl).

In contrast, AAV patients without clinical signs of renal involvement showed largely preserved renal function (eGFR 59–98 ml/min; serum creatinine 0.6–1.1 mg/dl).

**Table 1:** Demographic and clinical characteristics of patients with AAV, including patients with RPGN due to AAV and AAV patients without clinical signs of renal involvement. Proteinuria and hematuria were measured in spot urine samples; “normal” indicates values below the abnormal threshold (Proteinuria: <150 mg/l; Hematuria < 23 cells/µL)

Group	Age (years)	Sex	AAV Subtype	Proteinuria (mg/l)	Hematuria (cells/µl)	eGFR (ml/min)	Serum creatinine (mg/dl)
RPGN due to AAV	42	Female	MPA	839	1204	12	4,16
	62	Male	GPA	normal	normal	28	2,30
	81	Female	GPA	798	1098	58	0,97
AAV without clinical signs of renal involvement	65	Male	EGPA	normal	normal	89	0,97
	59	Female	EGPA	normal	48	59	1,04
	61	Female	GPA	normal	normal	98	0,60
	60	Female	MPA	normal	normal	70	1,10

Abbreviations: AAV, ANCA-associated vasculitis; RPGN, rapidly progressive glomerulonephritis; MPA, microscopic polyangiitis; GPA, granulomatosis with polyangiitis; EGPA, eosinophilic granulomatosis with polyangiitis; eGFR, estimated glomerular filtration rate

### Comparison of all patients with AAV and the healthy control group

**Fig. 2:**
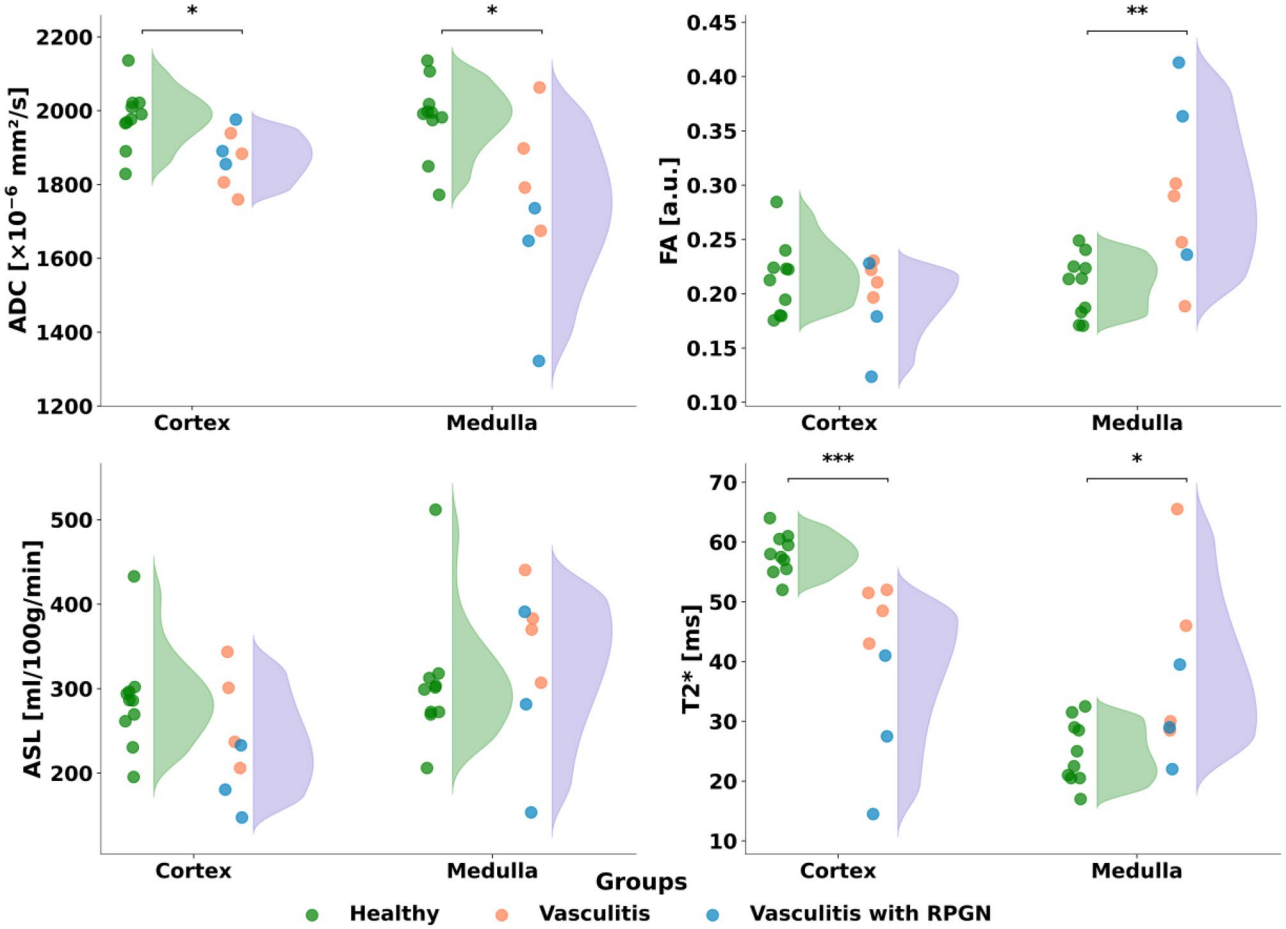
Comparison of renal MRI parameters between healthy controls, patients with AAV, and AAV patients with rapidly progressive glomerulonephritis (RPGN). Violin plots with overlaid individual data points show cortical and medullary values for ADC, FA, ASL, and T2*. Significant group differences are indicated by asterisks (*p < 0.05, **p < 0.01, ***p < 0.001).Abbreviations: ADC, apparent diffusion coefficient; FA, fractional anisotropy; ASL, arterial spin labeling; RPGN, rapidly progressive glomerulonephritis; AAV, ANCA-associated vasculitis

**Fig. 3:**
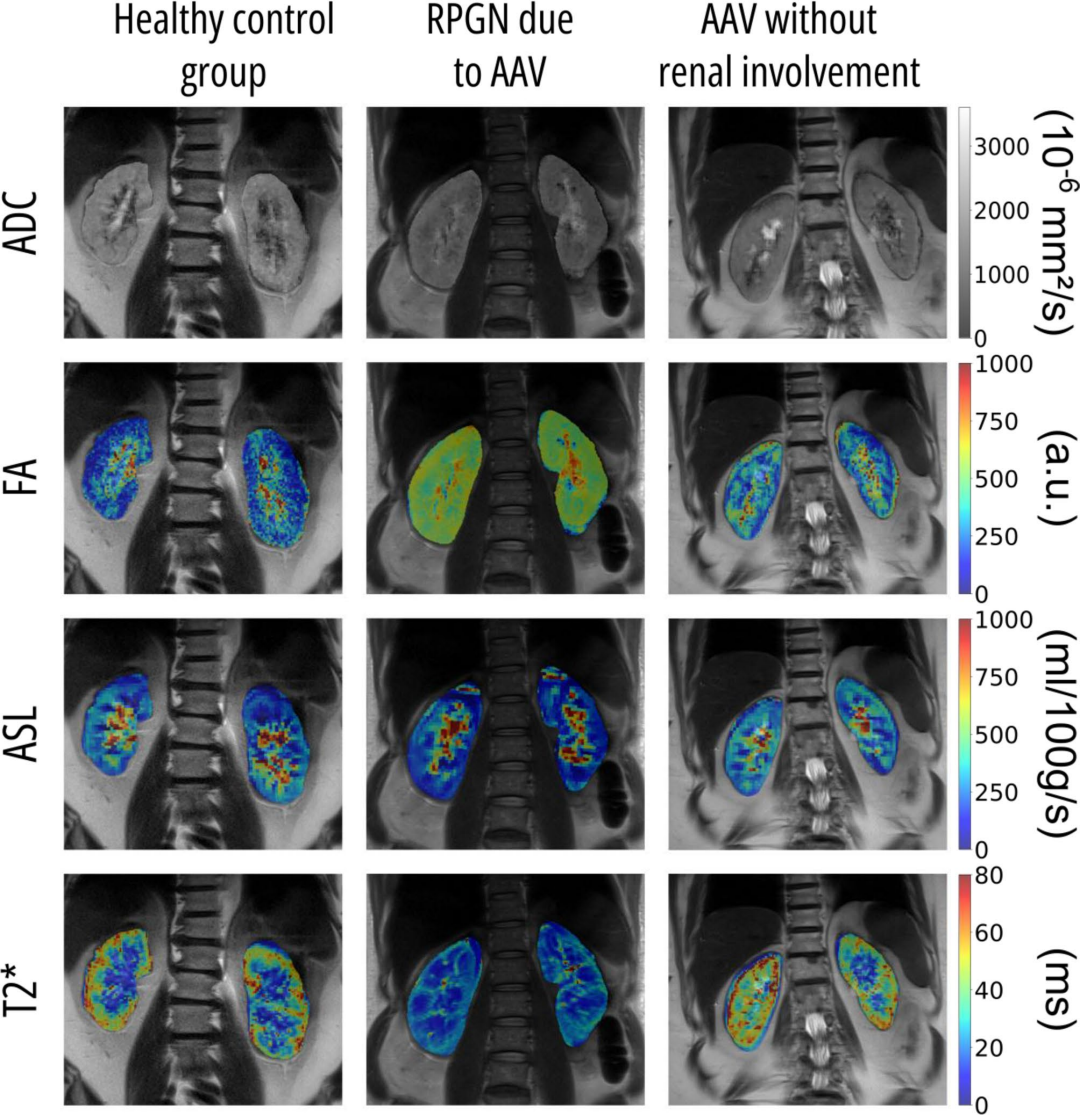
Representative renal MRI parameter maps (ADC, FA, ASL and T2*) in patients with ANCA-associated vasculitis and healthy controls. Cortical ADC values appeared markedly lower in both RPGN patients and those without confirmed renal involvement compared to healthy controls. Cortical perfusion, as assessed by ASL, was also reduced in both patient groups. Medullary FA values were higher in both patient groups compared to healthy controls. Additionally, cortical T2* values were lower than those observed in the healthy control group

Figure 2 shows multiparametric renal MRI data comparing patients with AAV and healthy controls. Across the AAV cohort, changes were observed in ADC, FA, ASL and tissue oxygenation (T2*). Figure 3 illustrates representative coronal renal MRI images and corresponding parametric maps for the different quantitative MRI techniques.

Table 2 summarizes the quantitative MRI parameters in healthy controls and AAV patients, stratified by renal cortex and medulla, including group sizes, mean values, standard deviations, and statistical significance: Compared to healthy controls, AAV patients showed significant differences in multiple MRI parameters. Cortical ADC values were significantly lower in patients than in controls (1873 ± 69 vs. 1981 ± 77 × 10-⁶ mm²/s, *p* = 0.01), and medullary ADC was also reduced (1733 ± 21 vs. 1982 ± 101 × 10-⁶ mm²/s, *p* = 0.02). Fractional anisotropy (FA) in the medulla was significantly higher in patients (0.29 ± 0.07) compared to controls (0.21 ± 0.03, *p* = 0.01). T2* values were markedly decreased in the cortex of AAV patients (39.7 ± 12.9 ms vs. 58 ± 3.3 ms, *p* = 0.001), whereas medullary T2* values were increased (37.2 ± 13.7 ms vs. 24.8 ± 5.0 ms, *p* = 0.04)

**Table 2:** Comparison of multiparametric MRI values between healthy controls and patients with AAV of cortical and medullary kidney regions. Mean and standard deviation are reported for each group. (* statistical significant = p<0,05)

Parameter	Region	*p*-value	*N* Healthy	*N* Disease	Mean Healthy ± SD	Mean Disease ± SD
**ADC** (10^-6^ mm^2^/s)	Cortex	0.01*	10	7	1980.9 +/-77.5	1872.857 +/-68.7
**ADC** (10^-6^ mm^2^/s)	Medulla	0.02*	10	7	1982.1 +/- 101.3	1733.286 +/-213.6
**ASL** (ml/100 g/s)	Cortex	0.23	10	7	285.5 +/-58.5	235.500 +/-62.8
**ASL** (ml/100 g/s)	Medulla	0.26	10	7	306.7 +/-75	332.357 +/-88.1
**FA** (a.u.)	Cortex	0.67	10	7	0.21 +/-0.03	0.199 +/-0.04
**FA** (a.u.)	Medulla	0.01*	10	7	0.21 +/-0.03	0.291 +/-0.07
T2* (ms)	Cortex	0.001*	10	7	58 +/-3.26	39.714 +/-12.9
T2* (ms)	Medulla	0.04*	10	7	24.8 +/-5.03	37.214 +/-13.68

Abbreviations: ADC, apparent diffusion coefficient; ASL, arterial spin labeling; FA, fractional anisotropy; T2*, effective transverse relaxation time; SD, standard deviation; a.u., arbitrary units

### Comparison between patients RPGN due to AAV and healthy controls

In the renal medulla, patients with biopsy proven RPGN due to AAV showed lower ADC values compared to the healthy volunteers (Table 3).

For the FA parameter, higher values were measured in the renal medulla of patients with RPGN compared to healthy volunteers.

For the ASL parameter, lower values were observed in the renal cortex of patients with RPGN compared to the healthy control group. No relevant differences were observed in the medulla.

Cortical T2* values were also lower in patients with RPGN compared to the healthy control group. In contrast, medullary values were higher in RPGN patients compared to healthy controls.

**Table 3:** Quantitative renal MRI parameters in patients with RPGN due to AAV and healthy controls. Data are presented as mean ± standard deviation for cortical and medullary regions

	ADC(10^-6^ mm^2^/s)		FA(a.u.)		ASL(ml/100g/s)		T2*(ms)	
	Cortex	Medulla	Cortex	Medulla	Cortex	Medulla	Cortex	Medulla
RPGN due to AAV(mean ± SD)	1907 +/-59	1588+/- 260	0.18+/-0.05	0.34+/- 0.09	187+/-48	275 +/- 131	28+/- 12	30+/-9
Healthy control group(mean ± SD)	1981+/- 89	1982+/- 109	0.21+/-0.04	0.21+/- 0.03	286+/-62	307+/-78	59+/-5	25+/-5

Abbreviations: ADC, apparent diffusion coefficient; FA, fractional anisotropy; ASL, arterial spin labeling perfusion; T2*, effective transverse relaxation time; AAV, ANCA-associated vasculitis; RPGN, rapidly progressive glomerulonephritis

### Comparison between patients with AAV without clinical signs of renal involvement and healthy controls

In patients with AAV without clinical signs of renal involvement such as proteinuria, erythrocyturia, or elevation of serum creatinine, lower ADC values were observed in the renal cortex and renal medulla compared to the healthy control group (Table 4).

For the ASL parameter, values were comparable between both groups.

For the FA parameter, higher values were observed in the renal medulla of patients with AAV without renal involvement compared to the healthy control group.

Similar to patients with RPGN, cortical T2* values were also lower in patients without previously confirmed renal involvement, while the medullary values in AAV patients tend to be rather elevated.

**Table 4:** Quantitative renal MRI parameters in patients AAV without renal involvement and healthy controls. Data are presented as mean ± standard deviation for cortical and medullary regions

	ADC (10^-6^ mm^2^/s)		FA (a.u.)		ASL (ml/100 g/s)		T2* (ms)	
	Cortex	Medulla	Cortex	Medulla	Cortex	Medulla	Cortex	Medulla
**Patients with AAV without clinical signs of renal involvement** (mean ± SD)	1847 +/-133	1785 +/- 184	0.22 +/-0.04	0.29 +/-0.05	272 +/-64	375 +/- 79	49 +/-5	37 +/-8
**Healthy control group** (mean ± SD)	1981 +/- 89	1982 +/- 109	0.21 +/-0.04	0.21 +/- 0.033	286 +/-62	307 +/-78	59 +/-5	25 +/-5

Abbreviations: ADC, apparent diffusion coefficient; FA, fractional anisotropy; ASL, arterial spin labeling perfusion; T2*, effective transverse relaxation time; AAV, ANCA-associated vasculitis

## Discussion

This study highlights the potential of functional MRI to detect renal alterations in patients with AAV. The differences observed in ADC, FA, T2* and ASL values between AAV patients and healthy controls suggest that these imaging biomarkers reflect structural and functional kidney impairment: Cortical ADC was reduced in AAV patients (1873 ± 69 vs. 1981 ± 77 × 10-⁶ mm²/s, *p* = 0.02), cortical T2* was markedly lower in AAV patients (39.7 ± 12.9 vs. 58.0 ± 3.3 ms, *p* = 0.001) and medullary FA was also elevated (0.29 ± 0.07 vs. 0.21 ± 0.03, *p* = 0.01). Interestingly, alterations were also detected in AAV patients without clinical manifestation of renal involvement, pointing to the possibility of early kidney alterations.

DWI is a non-invasive imaging technique that detects renal interstitial alterations, including fibrosis, inflammation, edema, and perfusion changes [7]. Previous studies have shown that the ADC value is positively correlated with the glomerular filtration rate, suggesting its potential as a biomarker for assessing renal function [22]. Acute and chronic renal failure also showed decreased ADC values compared with those of healthy volunteers [23]. In this study, patients with RPGN exhibited lower ADC values in the renal medulla compared to the healthy control group, whereas no differences were observed in the renal cortex. Previous studies have confirmed that in AAV, not only the glomeruli but also the tubuli and the tubular basement membrane (BM) can be affected, potentially leading to tubulointerstitial inflammation [24]. This could explain the reduced ADC values in the renal medulla. In patients with CKD, it has also been shown that ADC values decline more significantly in the renal medulla than in the cortex [7].

Previous studies on mpMRI have generally shown that FA values significantly decrease in kidney diseases [8]. In our study, higher medullary FA values were observed in patients with RPGN compared to the healthy control group. It is possible that during the acute inflammatory stage of vasculitis, tubular swelling in the medulla occurs, leading initially to an acceleration of fluid flow. A study also demonstrated that the Na⁺/H⁺ exchanger-3 is upregulated in glomerulonephritis, facilitating sodium uptake and proton secretion into the tubular system [18]. This could explain the increased directional proton flux during inflammatory responses in the kidney, potentially contributing to the elevation of the FA parameter.

Many studies report reproducibility of renal perfusion by ASL and lower ASL values in CKD patients compared with healthy subjects, which correlates with eGFR [25]. This reproducibility seems to be lower in the medulla than the cortex [11, 26]. This is also reflected in the study: ASL values in the cortex showed differences between patients with RPGN due to vasculitis and the healthy control group, with lower values in RPGN patients, whereas values in the medulla did not exhibit differences.

Studies indicate that renal hypoxia may be a key prognostic factor for the progression of chronic kidney disease [18]. BOLD-MRI has increasingly been utilized in recent years to evaluate alterations in renal oxygenation across various kidney diseases [18]. In this study, patients with RPGN due to AAV exhibited lower cortical T2* values compared to the healthy control group. The observed reduction in cortical T2* values in patients with AAV-associated RPGN may reflect altered cortical oxygenation related to inflammatory microvascular injury, impaired perfusion, and increased oxygen extraction [27].

Patients with AAV but without clinical manifestation of renal involvement were also examined and compared to the healthy control group. Consistent with the findings in patients with confirmed RPGN, differences were observed in BOLD imaging cortical as well as in medullary FA and ADC values. These findings suggest that these parameters may have the potential to detect early, clinically silent kidney alterations in patients with AAV. The ASL values did not show differences, suggesting preserved renal perfusion. In patients without clinical signs of renal involvement such as proteinuria or erythrocyturia, biopsy data are unfortunately not available due to ethical reasons. Biopsies might confirm structural changes that are already visible on mpMRI.

Future studies should focus on longitudinal assessments to evaluate the potential of functional MRI for disease monitoring of AAV over time. Repeated measurements could provide insights into disease progression and treatment response.

While MRI represents a cost factor, early detection of renal involvement could help prevent disease progression and reduce the need for more invasive or costly interventions. Delayed diagnosis may result in a prolonged disease course, leading to higher healthcare expenses due to extended hospital stays, more intensive treatments, or complications requiring dialysis. Therefore, implementing MRI as a non-invasive monitoring tool could potentially improve patient outcomes while optimizing healthcare resources.

The low number of RPGN patients represents a limitation of this study. Given the incidence of RPGN of only 7 cases per 1,000,000 inhabitants, an increase in patient numbers would likely only be feasible through a multicenter study [3].

Given the small cohort size and the heterogeneity of AAV subtypes, potential subtype-specific differences in renal involvement and their impact on MR parameters cannot be ruled out, which may have influenced the observed results.

Another limitation of this study is the relatively low number of b-values used for diffusion-weighted imaging. However, the MRI protocol was designed to be feasible within a clinically acceptable scanning time. Despite this constraint, the acquired diffusion-weighted sequences were still analyzable and provided valuable insights into renal function.

Baseline characteristics such as age, blood pressure, and diabetes are known to influence renal perfusion and may therefore represent relevant confounding factors. These variables were not specifically controlled for in the present analysis. Therefore, a potential influence on the observed perfusion results cannot be excluded.

In conclusion this study demonstrates that functional MRI may provide preliminary evidence for detecting renal alterations in ANCA-associated vasculitis. Differences in ADC, FA, T2* and ASL values between RPGN patients and healthy controls suggest that these parameters reflect structural and functional kidney impairment. Notably, imaging markers were also altered in patients without confirmed renal involvement, indicating potential early kidney damage.

Overall, these findings support the feasibility of functional renal MRI as a non-invasive tool for studying and monitoring vasculitis-related kidney disease, while further research is needed to confirm its diagnostic value.

## Data Availability

The datasets used and/or analysed during the current study are available from the corresponding author on reasonable request.
